# Analysis of peripheral blood T-cell levels for predicting immunotherapy response sensitivity in squamous cell carcinoma of the head and neck

**DOI:** 10.3389/fmolb.2026.1896236

**Published:** 2026-08-06

**Authors:** Nuan Li, Lifei Feng, Gaofei Yin, Shuo Ding, Wei Guo

**Affiliations:** 1 Beijing Tongren Hospital, Capital Medical University, Beijing, China; 2 Department of Otolaryngology-Head and Neck Surgery, Key Laboratory of Otolaryngology-Head and Neck Surgery, Ministry of Education, Beijing Tongren Hospital, Capital Medical University, Beijing, China

**Keywords:** CD28, head and neck squamous cell carcinoma (HNSCC), immunotherapy, PCR, PD-L1, peripheral blood immune cells

## Abstract

**Objective:**

To investigate the correlation between peripheral blood immune cell subsets and pathological response to neoadjuvant therapy in locally advanced head and neck squamous cell carcinoma (HNSCC), and to evaluate their predictive value.

**Methods:**

A total of 57 patients with locally advanced HNSCC were retrospectively enrolled. Patients were divided into a chemotherapy group (TPF regimen, n = 25) and a chemoimmunotherapy group (TPK regimen, n = 32). Peripheral blood percentages and absolute counts of CD4^+^CD25^+^CD127low Treg cells, CD3^+^PD-1^+^, CD3^+^CD28^+^, CD4^+^PD-1^+^, CD4^+^CD28^+^, CD8^+^PD-1^+^, and CD8^+^CD28^+^ cells, as well as CPS scores, were collected before and after treatment. Primary tumor and lymph node responses were assessed by RECIST 1.1 and histopathology.

**Results:**

In the chemoimmunotherapy group, primary tumor and dual pCR rates were 75.0% (24/32) and 37.0% (10/27), respectively; subsequent surgery was significantly reduced. Post-chemoimmunotherapy, CD3^+^CD28^+^ (P = 0.014) and CD8^+^CD28^+^ (P = 0.034) percentages were lower in the primary tumor pCR group vs. non-pCR; in the pCR group, CD3^+^CD28^+^ (P = 0.026) and CD8^+^CD28^+^ (P = 0.041) percentages significantly declined from baseline. In lymph node pCR, similar trends were observed, with an additional decrease in CD4^+^CD28^+^ percentage (P = 0.030). In dual pCR, CD3^+^CD28^+^ percentage trended downward after treatment (P = 0.052). In the chemotherapy group, post-treatment Treg count (P = 0.013) and CD3^+^CD28^+^ percentage (P = 0.038) were lower in primary tumor pCR vs. non-pCR, with no significant associations at nodal or composite levels. Representative cases confirmed that benefit was associated with a sustained decline in peripheral CD3^+^CD28^+^ percentage, whereas non-beneficiaries showed stable or rebounding levels.

**Conclusion:**

Chemoimmunotherapy can induce a significant decrease in peripheral blood CD28^+^ T cell subsets in HNSCC patients, and this dynamic change is closely associated with pathological complete response in the primary tumor, lymph nodes, and dual sites, with its predictive performance being superior to that of traditional CPS scoring. The migratory response of peripheral blood activated T cells holds promise as a novel non-invasive biomarker to guide individualized neoadjuvant therapy in locally advanced HNSCC.

## 1 Introduction

Head and neck squamous cell carcinoma (HNSCC) is one of the six most common cancers worldwide and is characterized by a high incidence, low screening rates, poor prognosis, and a high disability rate, imposing a heavy burden on both society and families (Sung et al., 2021). Because of its insidious early symptoms and the lack of effective screening tools, approximately 60% of patients present with locally advanced disease (stage III-IVB) at initial diagnosis, with extensive tumor invasion into structures of the upper aerodigestive tract such as the oropharynx, larynx, or hypopharynx, making treatment extremely challenging (Chow, 2020). For locally advanced HNSCC, preserving physiological functions such as phonation, swallowing, and respiration to the greatest extent possible while ensuring survival represents the central goal of current clinical management. A series of landmark studies have established the role of induction chemotherapy, concurrent chemoradiotherapy, and neoadjuvant therapy in sequential organ-preservation strategies. For example, long-term follow-up of the RTOG 91–11 trial demonstrated that cisplatin-based induction chemotherapy followed by radiotherapy for locally advanced laryngeal cancer achieves survival rates comparable to total laryngectomy while enabling laryngeal preservation in a substantial proportion of patients (Forastiere et al., 2013). In recent years, immune checkpoint inhibitors have reshaped the treatment landscape of HNSCC and have been moved forward into the preoperative neoadjuvant setting. A single-arm phase II study showed that neoadjuvant pembrolizumab monotherapy in resectable locally advanced HNSCC yielded a major pathological response (MPR, ≤10% residual viable tumor) rate of 44% and a pathological complete response (pCR) rate of 22% (Uppaluri et al., 2020); neoadjuvant immunotherapy combined with chemotherapy has demonstrated even deeper pathological responses, with a pCR rate of 37.0% reported for camrelizumab plus chemotherapy (Zhang et al., 2022). These data strongly indicate that neoadjuvant immunotherapy can effectively induce tumor regression and achieve clinical downstaging, thereby creating more favorable conditions for organ preservation and functional maintenance.

However, the efficacy of neoadjuvant immunotherapy exhibits substantial interindividual variability. Even with the combination regimens that yield the highest pathological response rates, more than half of patients fail to achieve a major pathological response, and some patients even experience disease progression (Wang et al., 2023; Wise-Draper et al., 2022). How to accurately identify patients who are likely to benefit before the initiation of neoadjuvant therapy is a critical challenge that needs to be urgently addressed in clinical practice. Currently, the Combined Positive Score (CPS) is the only companion diagnostic biomarker widely used for immunotherapy decision-making in HNSCC, but its predictive performance in the neoadjuvant setting remains controversial. Uppaluri et al. found that pathological response rates to neoadjuvant pembrolizumab did not differ significantly between patients with CPS ≥1 and those with CPS <1, suggesting that tumor PD-L1 expression has limited predictive value in this context (Uppaluri et al., 2020). Moreover, tissue-based biomarkers such as CPS, tumor mutational burden (TMB), and immune gene expression profiling also face considerable challenges, including intratumoral heterogeneity, sampling-related trauma, and the inability to perform dynamic monitoring. Therefore, identifying a minimally invasive and repeatedly obtainable biomarker that can accurately predict the response to neoadjuvant immunotherapy is essential for advancing personalized precision therapy.

Peripheral blood, as the most accessible liquid biopsy specimen, contains abundant information on circulating immune cells. The core mechanism of anti-PD-1 immunotherapy involves reactivating exhausted T cells by blocking PD-1/PD-L1 signaling, and T cell subsets in peripheral blood include both effector cells capable of recognizing tumor antigens and reflect the systemic immune status of the host. Among these subsets, CD8^+^ T cells are the principal executors of tumor killing, and the level of PD-1 expression on their surface is closely associated with T cell activation, exhaustion, and reinvigoration potential. Studies in multiple solid tumors have demonstrated that the baseline abundance of peripheral blood CD8^+^PD-1^+^ T cells and their proliferative dynamics early during treatment can independently predict clinical benefit from anti-PD-1 therapy (Kim et al., 2019). However, a systematic evaluation of the predictive value of various peripheral blood T cell subsets (including CD3^+^PD-1^+^, CD4^+^PD-1^+^, CD8^+^PD-1^+^ cells, et al.) in the neoadjuvant immunotherapy setting, as well as a direct comparison with the traditional tissue-based biomarker CPS, remains lacking.

Therefore, this study retrospectively enrolled 57 patients with HNSCC and measured peripheral blood immune cell subset levels before and after treatment, combined with CPS scores, to analyze their correlation with neoadjuvant treatment response. The aim was to provide new evidence and strategies for the non-invasive prediction of neoadjuvant immunotherapy efficacy in HNSCC.

## 2 Materials and methods

### 2.1 Study subjects

This was a single-center, retrospective case series that consecutively enrolled 57 patients with locally advanced HNSCC who were initially diagnosed at Beijing Tongren Hospital, Capital Medical University between May 2025 and May 2026. The inclusion criteria were as follows: (1) histopathologically confirmed HNSCC; (2) complete clinical history, laboratory, imaging, and pathological data available. The exclusion criteria were as follows: (1) concurrent other malignancies or distant metastasis confirmed by comprehensive clinical evaluation; (2) recurrent disease; (3) occurrence of grade ≥3 serious adverse events during neoadjuvant therapy leading to treatment discontinuation or regimen modification and failure to complete the planned treatment course; (4) active autoimmune disease or prior immunosuppressive therapy; (5) refusal to participate in follow-up or incomplete pathological response assessment due to other uncontrollable reasons. All patients received standardized neoadjuvant treatment in accordance with current clinical guidelines and underwent regular follow-up (Colevas et al., 2025). All examinations and treatments were conducted in compliance with the Declaration of Helsinki.

### 2.2 Treatment regimens and data collection

Based on the neoadjuvant treatment received, patients were divided into two groups: the chemotherapy group received the TPF regimen (paclitaxel on Day 1, nedaplatin on Days 2–4, and fluorouracil on Days 2–6, repeated every 3 weeks per cycle), and the chemoimmunotherapy group received the TPK regimen (paclitaxel on Day 1, nedaplatin on Days 1–3, and pembrolizumab on Day 4, repeated every 3 weeks per cycle). Venous whole blood samples (1 mL) were collected in the morning after overnight fasting at two time points: 1 day before the initiation of treatment and 1 day before surgical evaluation. Samples were anticoagulated with EDTA-K_2_, stored at room temperature (20 °C–25 °C), and analyzed by flow cytometry within 24 h of collection. The following seven T-cell subsets were measured as percentages and absolute counts: CD4^+^CD25^+^CD127LOW Treg cells, CD3^+^PD-1^+^, CD3^+^CD28^+^, CD4^+^PD-1^+^, CD4^+^CD28^+^, CD8^+^PD-1^+^, and CD8^+^CD28^+^ (Supplementary Materials and Supplementary Table 2 for details).

After completion of neoadjuvant therapy, all patients underwent surgical treatment. Surgical specimens were independently evaluated by two experienced head and neck pathologists, who were blinded to the patients’ treatment group assignment and peripheral blood immune cell data. Standardized comprehensive tumor bed sampling was performed, with at least one section taken per centimeter of the primary tumor bed and all regional lymph nodes individually submitted for histological examination. Primary tumor pathological complete response (pCR) was defined as the complete absence of viable tumor cells in the primary tumor bed, with only treatment-related changes such as necrosis, fibrosis, and histiocytic aggregates present. Lymph node pCR was defined as the absence of viable tumor cells in all submitted lymph nodes. Non-pCR was defined as the presence of any viable tumor cells in either the primary tumor or lymph node specimens. Dual pCR was defined as achieving pCR simultaneously in both the primary tumor and lymph nodes. Because the percentage of residual viable tumor was not routinely reported in the original pathological reports, no stratified analysis for major pathological response (MPR) was performed in this study.

Clinical data were collected for all 57 patients, encompassing the following aspects: demographic information (sex, age, smoking history, and alcohol history), baseline tumor characteristics (primary tumor site, T stage, N stage, and clinical stage), subsequent surgical treatment, pathological data (CPS score, histological type, and degree of differentiation), and peripheral blood laboratory test data before and after treatment. CPS (Combined Positive Score) was defined as the ratio of the total number of PD-L1-positive cells (tumor cells and immune cells) to the total number of viable tumor cells, multiplied by 100, and was independently assessed by two pathologists via immunohistochemistry.

## 3 Statistical analysis

Data were collected and summarized using Microsoft Excel 2019, and statistical analyses were performed using SPSS Statistics 23.0. Normally distributed continuous variables are presented as mean ± standard deviation, and between-group comparisons were performed using one-way ANOVA for multi-group comparisons or paired/independent-samples t-tests for two-group comparisons, as appropriate. Non-normally distributed continuous variables are presented as median (first quartile, third quartile), and between-group comparisons were conducted using the Mann-Whitney U test for independent samples or the Wilcoxon signed-rank test for paired samples. All comparisons of immune indices were performed using two-sided tests, and the significance level was uniformly corrected for multiple comparisons using the Dunn-Bonferroni method. Categorical variables were compared between groups using the chi-square test or Fisher’s exact test. A significance level of 0.05 was set for all analyses.

## 4 Results

### 4.1 Descriptive analysis of baseline characteristics and evaluation of treatment response

A total of 57 patients were enrolled in this study, including 53 males (93.0%) and four females (7.0%), with a mean age of 62.3 ± 8.6 years. Thirty-two patients (56.1%) received chemoimmunotherapy (TPK regimen) and 25 (43.9%) received chemotherapy alone (TPF regimen). The distribution of primary tumor sites was as follows: hypopharyngeal carcinoma in 29 patients (50.9%), laryngeal carcinoma in 16 (28.1%), oropharyngeal carcinoma in 10 (17.5%), and other sites in 2 (3.5%) (Table 1). After comprehensive assessment of their general condition and baseline tumor characteristics, all patients confirmed their neoadjuvant treatment regimen based on personal preference and received 2-4 cycles of treatment. Following treatment completion, contrast-enhanced neck CT and MRI examinations were performed, and imaging evaluation was conducted according to the Response Evaluation Criteria in Solid Tumors (RECIST 1.1), with subsequent histopathological confirmation using surgical specimens.

**TABLE 1:** Comparison of baseline and surgical characteristics between treatment groups.

Item	Classification	Chemoimmunotherapy		Chemotherapy		Statistical	*P*
		n	Statistical	n	Statistical		
Sex	Male	30	52.6%	23	40.4%	χ^2^ = 0.066	1.000
	Famale	2	3.5%	2	3.5%		
Age (years)	-	32	60.56 ± 1.62	25	64.52 ± 1.49	t = −1.751	0.086
Smoking history	Yes	16	28.1%	6	10.5%	χ^2^ = 4.003	0.058
	No	16	28.1%	19	33.3%		
Alcohol history	Yes	12	21.1%	10	17.5%	χ^2^ = 0.037	1.000
	No	20	35.1%	15	26.3%		
Cycles	2	19	33.3%	19	33.3%	χ^2^ = 2.651	0.428
	3	11	19.3%	6	10.5%		
	4	2	3.5%	0	0.0%		
Primary site	Hypopharynx	17	29.8%	12	21.1%	χ^2^ = 1.881	0.617
	Larynx	7	12.3%	9	15.8%		
	Oropharynx	7	12.3%	3	5.3%		
	Others	1	1.8%	1	1.8%		
Esophageal invasion	No	30	52.6%	24	42.1%	χ^2^ = 0.143	1.000
	Yes	2	3.5%	1	1.8%		
T Stage	1	1	1.8%	1	1.8%	Z = −1.001	0.317
	2	5	8.8%	7	12.3%		
	3	13	22.8%	9	15.8%		
	4	13	22.8%	8	14.0%		
N stage	0	6	10.5%	8	14.0%	Z = −0.300	0.764
	1	1	1.8%	0	0.0%		
	2	24	42.1%	14	24.6%		
	3	1	1.8%	3	5.3%		
Clinical stage	II	1	1.8%	2	3.5%	Z = −0.345	0.730
	III	7	12.3%	5	8.8%		
	IV	24	42.1%	18	31.6%		
Differentiation	Poorly	8	14.0%	13	22.8%	χ^2^ = 4.470	0.107
	Moderately	19	33.3%	9	15.8%		
	Unspecified	5	8.8%	3	5.3%		
CPS	-	32	20.0 (5.0.40.0)	25	15.0 (4.0.50.0)	Z = −0.331	0.741
P16	Negative	25	45.5%	20	36.4%	χ^2^ = 0.066	1.000
	Positive	6	10.9%	4	7.3%		
Surgical approach	Minimally	29	50.9%	16	28.1%	χ^2^ = 5.986	0.022
	Open	3	5.3%	9	15.8%		
Neck dissection	No	11	19.3%	8	14.0%	χ^2^ = 0.143	1.000
	Unilateral	10	17.5%	9	15.8%		
	Bilateral	11	19.3%	8	14.0%		

**FIGURE 1:**
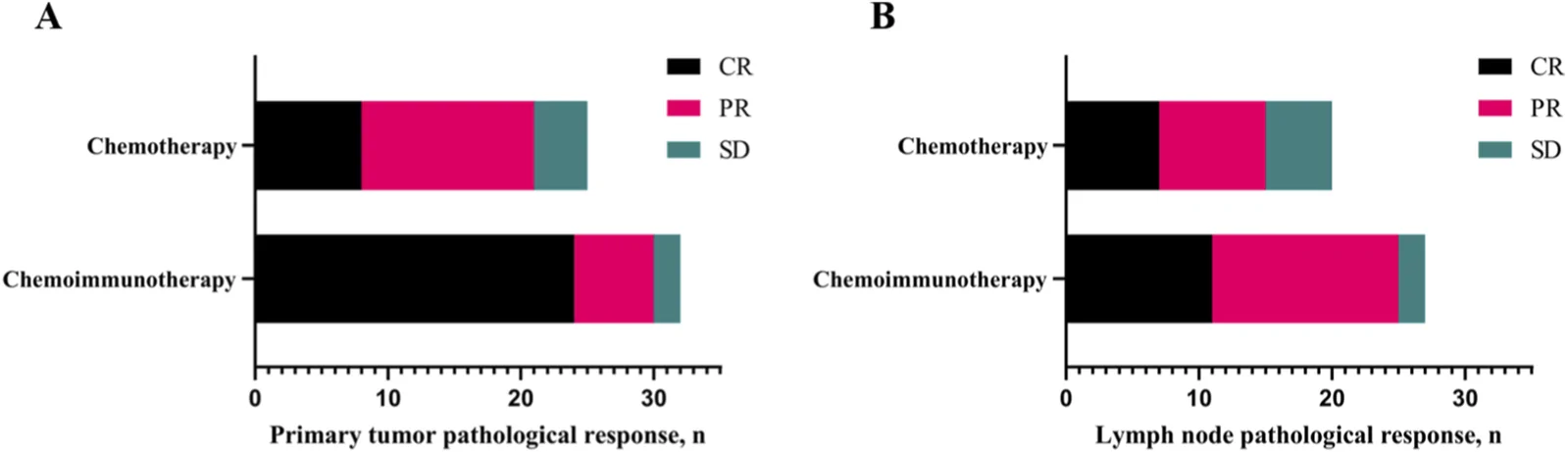
Pathological response of primary tumor and lymph nodes by treatment group. **(A)** Pathological response of the primary tumor. **(B)** Pathological response of lymph nodes.

The evaluation results showed that for the primary tumor, 32 patients achieved a complete response (CR), 19 a partial response (PR), and 6 stable diseases (SD), with a statistically significant difference in response between the two treatment groups (χ^2^ = 10.467, P = 0.003, Figure 1A). Among patients with evaluable lymph node metastases (n = 47), 18 achieved nodal CR, 22 nodal PR, and 7 nodal SD, with no statistically significant difference between the groups (χ^2^ = 2.708, P = 0.227, Figure 1B). Additionally, patients in the chemoimmunotherapy group subsequently underwent significantly reduced surgical resection extent and had a significantly higher proportion of minimally invasive surgery, with the differences being statistically significant (Table 1).

### 4.2 Correlation analysis between primary tumor response and peripheral blood immune cells in different treatment groups

Before conducting between-group comparisons, the comparability of pretreatment baseline characteristics was first confirmed. No statistically significant differences were observed between the chemoimmunotherapy group and the chemotherapy-only group, or between the pCR and non-pCR subgroups within each treatment group, in terms of demographic characteristics, tumor stage, HPV infection status, CPS score, and all 13 peripheral blood immune cell parameters (all P > 0.05). This effectively controlled for sampling bias and confounding effects arising from differences in pretreatment immune status, thereby providing a balanced baseline for the subsequent efficacy-related analyses.

Given the limited number of patients with stable disease (SD) in each treatment group, patients were reclassified based on histopathological results into a pathological complete response (pCR) group and a non-pCR group for analysis. A subgroup analysis was first conducted within the chemoimmunotherapy group (n = 32), comparing differences in the CPS score and 13 peripheral blood immune cell parameters between the pCR group (n = 24) and the non-pCR group (n = 8). The latter encompassed the percentages and absolute counts of CD4^+^CD25^+^CD127Low cells (Treg cells), CD3^+^PD-1^+^, CD3^+^CD28^+^, CD4^+^PD-1^+^, CD4^+^CD28^+^, CD8^+^PD-1^+^, and CD8^+^CD28^+^ cells. The analysis included pretreatment baseline levels, post-treatment levels, and the percent change from baseline [(post-treatment - pretreatment) / pretreatment × 100%].

The results showed that within the chemoimmunotherapy group, no statistically significant differences were observed in the baseline characteristics of peripheral blood immune cells between the pCR and non-pCR groups. After neoadjuvant treatment, the percentage of CD3^+^CD28^+^ cells (P = 0.014) and the percentage of CD8^+^CD28^+^ cells (P = 0.034) were significantly lower in the pCR group than in the non-pCR group. When dynamic evolution was assessed using percent change from baseline, the percentage of CD3^+^CD28^+^ cells decreased significantly from pretreatment in the pCR group (P = 0.026), and the percentage of CD8^+^CD28^+^ cells also decreased significantly (P = 0.041) (Table 2).

**TABLE 2:** Peripheral blood immune indices in the chemoimmunotherapy group by primary tumor pathological response.

Category	Item	Chemoimmunotherapy		Statistical	*P*
		pCR (n = 24)	Non-pCR (n = 8)		
Pre-treatment	CPS	20.00 (5.00.67.50)	15.00 (5.75.35.00)	Z = −0.546	0.593
Pre-treatment	Treg	3.13 (2.55.4.52)	3.59 (2.36.5.11)	Z = −0.392	0.717
	CD3+PD1+(%)	30.95 ± 4.92	32.53 ± 8.47	t = −0.500	0.630
	CD3^+^CD28+(%)	66.13 ± 11.19	64.50 ± 14.33	t = 0.333	0.742
	CD4+PD1+(%)	26.36 (23.25.32.13)	28.15 (20.67.37.65)	Z = −0.087	0.949
	CD4^+^CD28+(%)	86.44 (76.02.91.00)	82.47 (77.45.89.04)	Z = −0.522	0.623
	CD8+PD1+(%)	33.58 (26.56.37.67)	36.27 (25.50.47.06)	Z = 0.609	0.564
	CD8^+^CD28+(%)	43.89 ± 14.13	43.86 ± 14.94	t = 0.005	0.996
	CD3+PD1+	294.00 (204.50,475.75)	277.50 (121.25,638.25)	Z = −0.348	0.749
	CD3^+^CD28^+^	778.50 (431.75,994.00)	567.00 (361.00,1649.75)	Z = −0.305	0.782
	CD4+PD1+	167.00 (123.75,261.75)	160.50 (84.00,381.25)	Z = −0.261	0.815
	CD4^+^CD28^+^	518.50 (265.75,868.50)	453.50 (291.25,1362.25)	Z = −0.065	0.949
	CD8+PD1+	110.50 (64.50,171.00)	110.50 (32.00,211.50)	Z = −0.261	0.815
	CD8^+^CD28^+^	147.50 (100.25,215.75)	116.00 (44.00,428.50)	Z = −0.435	0.685
Post-treatment	Treg	4.44 ± 1.99	3.47 ± 1.93	t = 1.200	0.239
	CD3+PD1+(%)	0.68 (0.43.1.33)	0.66 (0.56.0.89)	Z = −0.283	0.782
	CD3^+^CD28+(%)	63.01 ± 11.57	72.31 ± 7.12	t = −2.694	0.014
	CD4+PD1+(%)	0.43 (0.28.0.80)	0.38 (0.26.0.53)	Z = −0.631	0.535
	CD4^+^CD28+(%)	80.56 (67.45.89.74)	86.92 (68.33.92.37)	Z = −1.088	0.293
	CD8+PD1+(%)	0.82 (0.57.1.31)	0.90 (0.13.1.26)	Z = −0.588	0.564
	CD8^+^CD28+(%)	43.47 ± 17.48	52.74 ± 6.04	t = −2.228	0.034
	CD3+PD1+	6.00 (3.25.10.00)	5.50 (5.00.8.75)	Z = −0.022	0.983
	CD3^+^CD28^+^	462.00 (351.25,748.00)	680.50 (497.00,882.50)	Z = 1.523	0.135
	CD4+PD1+	2.00 (2.00.3.75)	2.00 (1.25.2.75)	Z = 0.769	0.480
	CD4^+^CD28^+^	342.50 (274.00,488.75)	470.00 (309.00,666.50)	Z = −1.175	0.254
	CD8+PD1+	2.00 (1.00.3.75)	2.00 (0.25.3.00)	Z = −0.666	0.535
	CD8^+^CD28^+^	111.50 (58.75,165.50)	127.50 (90.75,207.50)	Z = −0.871	0.404
% change	Treg	0.33 ± 0.45	0.03 ± 0.79	t = 1.361	0.184
	CD3+PD1+(%)	−0.98 (-0.99,-0.96)	−0.98 (-0.98,-0.97)	Z = −0.392	0.717
	CD3^+^CD28+(%)	−0.03 ± 0.18	0.18 ± 0.33	t = −2.342	0.026
	CD4+PD1+(%)	−0.98 (-0.99,-0.97)	−0.99 (-0.99,-0.98)	Z = −0.783	0.454
	CD4^+^CD28+(%)	−0.023 (-0.12.0.04)	0.06 (-0.18.0.13)	Z = −1.044	0.313
	CD8+PD1+(%)	−0.97 (-0.98,-0.95)	−0.98 (-1.00,-0.96)	Z = −0.957	0.357
	CD8^+^CD28+(%)	0.01 (-0.16.0.13)	0.17 (0.01.0.46)	Z = −2.045	0.041
	CD3+PD1+	−0.98 (-0.99,-0.97)	−0.98 (-0.99,-0.95)	Z = −0.479	0.654
	CD3^+^CD28^+^	−0.24 (-0.53.0.21)	0.02 (-0.48.0.38)	Z = −0.653	0.535
	CD4+PD1+	−0.99 (-0.99,-0.98)	−0.99 (-0.99,-0.97)	Z = −0.218	0.848
	CD4^+^CD28^+^	−0.20 (-0.54.0.25)	−0.19 (-0.51.0.34)	Z = −0.348	0.749
	CD8+PD1+	−0.98 (-0.99,-0.97)	−0.99 (-1.00,-0.95)	Z = −0.566	0.593
	CD8^+^CD28^+^	−0.39 (-0.51.0.21)	0.19 (-0.61.1.13)	Z = −1.132	0.273

The same analyses were performed in the chemotherapy group (n = 25). The pretreatment baseline characteristics were comparable between the two groups. After chemotherapy, the absolute count of peripheral blood Treg cells (P = 0.013) and the percentage of CD3^+^CD28^+^ cells (P = 0.038) were significantly lower in the pCR group than in the non-pCR group. Furthermore, the percentage of CD3^+^CD28^+^ cells in the pCR group decreased significantly from baseline after chemotherapy (P = 0.013). These findings were largely consistent with the trends observed in the chemoimmunotherapy group (Table 3).

**TABLE 3:** Peripheral blood immune indices in the Chemotherapy group by primary tumor pathological response.

Category	Item	Chemotherapy		Statistical	*P*
		pCR (n = 8)	Non-pCR (n = 17)		
Pre-treatment	CPS	17.50 (4.25.33.75)	15.00 (4.00.50.00)	Z = −0.322	0.754
Pre-treatment	Treg	3.37 ± 0.90	3.83 ± 2.38	t = −0.531	0.601
	CD3+PD1+(%)	30.08 ± 9.06	31.04 ± 7.15	t = −0.288	0.776
	CD3^+^CD28+(%)	67.22 ± 12.08	61.97 ± 17.30	t = 0.770	0.449
	CD4+PD1+(%)	28.68 ± 10.39	30.65 ± 6.71	t = −0.576	0.570
	CD4^+^CD28+(%)	85.10 (73.50.89.39)	83.72 (66.68.87.74)	Z = −0.583	0.588
	CD8+PD1+(%)	31.20 ± 12.13	33.88 ± 11.51	t = −0.335	0.740
	CD8^+^CD28+(%)	42.01 ± 18.21	39.10 ± 17.81	t = 0.379	0.708
	CD3+PD1+	367.00 (180.00,342.50)	256.00 (196.50,315.50)	Z = −0.175	0.887
	CD3^+^CD28^+^	666.50 (545.25,736.75)	508.00 (403.00,735.00)	Z = −1.107	0.288
	CD4+PD1+	152.00 (117.75,233.00)	155.00 (113.00,197.50)	Z = −0.087	0.932
	CD4^+^CD28^+^	535.00 (387.00,621.25)	393.00 (309.00,602.50)	Z = −1.107	0.288
	CD8+PD1+	88.88 ± 52.89	104.94 ± 61.01	t = −0.639	0.529
	CD8^+^CD28^+^	125.00 (73.50,149.00)	94.00 (56.50,152.00)	Z = −0.204	0.842
Post-treatment	Treg	2.34 (1.77.3.07)	3.42 (2.99.4.11)	Z = −2.448	0.013
	CD3+PD1+(%)	27.90 ± 15.27	32.45 ± 6.59	t = −0.809	0.441
	CD3^+^CD28+(%)	55.56 ± 18.02	70.69 ± 15.14	t = −2.196	0.038
	CD4+PD1+(%)	26.68 ± 15.01	31.20 ± 6.88	t = −0.812	0.439
	CD4^+^CD28+(%)	69.34 ± 20.36	85.23 ± 8.02	t = −2.131	0.066
	CD8+PD1+(%)	28.72 ± 16.48	34.16 ± 9.27	t = −1.063	0.299
	CD8^+^CD28+(%)	33.04 ± 14.14	47.71 ± 23.72	t = −1.609	0.121
	CD3+PD1+	228.00 (102.50,409.50)	225.00 (144.00,297.00)	Z = −0.058	0.977
	CD3^+^CD28^+^	440.50 (214.75,729.75)	446.00 (308.50,725.00)	Z = −0.350	0.754
	CD4+PD1+	150.00 (76.75,252.75)	128.00 (99.00,175.50)	Z = −0.466	0.669
	CD4^+^CD28^+^	392.00 (174.50,614.75)	319.00 (251.00,514.50)	Z = −0.350	0.754
	CD8+PD1+	55.00 (18.00,141.25)	64.00 (33.50,104.00)	Z = −0.175	0.887
	CD8^+^CD28^+^	65.00 (28.25,149.50)	94.00 (42.50,157.50)	Z = −0.758	0.475
% change	Treg	−0.30 (-0.55,-0.01)	−0.10 (-0.49.0.25)	Z = −0.874	0.406
	CD3+PD1+(%)	−0.11 ± 0.41	0.10 ± 0.33	t = −1.352	0.189
	CD3^+^CD28+(%)	−0.19 (0.40.0.05)	0.06 (-0.00.0.46)	Z = −2.447	0.013
	CD4+PD1+(%)	−0.02 (-0.19.0.15)	0.02 (-0.07.0.10)	Z = −0.408	0.711
	CD4^+^CD28+(%)	−0.13 (-0.31,-0.00)	0.02 (-0.04.0.28)	Z = −2.388	0.016
	CD8+PD1+(%)	−0.05 (0.49.0.15)	−0.68 (-0.13.0.20)	Z = −0.816	0.440
	CD8^+^CD28+(%)	−0.09 (-0.46.0.18)	0.21 (-0.03.0.46)	Z = −1.573	0.124
	CD3+PD1+	−0.01 ± 0.67	−0.03 ± 0.48	t = 0.102	0.919
	CD3^+^CD28^+^	−0.15 ± 0.52	0.12 ± 0.67	t = −0.984	0.335
	CD4+PD1+	0.02 ± 0.66	0.02 ± 0.52	t = −0.011	0.991
	CD4^+^CD28^+^	−0.21 (-0.50.0.19)	−0.13 (-0.29.0.59)	Z = −1.107	0.288
	CD8+PD1+	−0.08 ± 0.71	−0.18 ± 0.41	t = 0.458	0.651
	CD8^+^CD28^+^	−0.30 (-0.56.0.52)	−0.26 (-0.44.0.32)	Z = −0.291	0.798

### 4.3 Correlation analysis between lymph node response and peripheral blood immune cells in different treatment groups

Among patients with evaluable cervical lymph node metastases (27 in the chemoimmunotherapy group and 20 in the chemotherapy group), patients were divided into a pCR group and a non-pCR group according to pathological lymph node response. In the chemoimmunotherapy group, there were 11 patients in the nodal pCR group and 16 in the non-pCR group; the pretreatment baseline characteristics of peripheral blood immune cells were comparable between the two groups. After treatment, the percentage of CD3^+^CD28^+^ cells (P = 0.008) and the percentage of CD8^+^CD28^+^ cells (P = 0.039) were significantly lower in the nodal pCR group than in the non-pCR group. Within-group comparisons showed that the percentage of CD3^+^CD28^+^ cells in the pCR group decreased significantly from pretreatment (P = 0.044); in addition, the percentage of CD4^+^CD28^+^ cells also decreased significantly (P = 0.030) (Table 4). In the chemotherapy group, subgroup analysis based on lymph node pathological response did not reveal any statistically significant differences in peripheral blood immune cell parameters (Supplementary Table 1).

**TABLE 4:** Peripheral blood immune indices in the chemoimmunotherapy group by lymph node pathological response.

Category	Item	Chemoimmunotherapy		Statistical	*P*
		pCR (n = 11)	Non-pCR (n = 16)		
Pre-treatment	CPS	20.00 (5.00.80.00)	20.00 (5.25.40.00)	Z = 0.323	0.753
Pre-treatment	Treg	3.28 (3.08.3.99)	3.29 (2.53.4.70)	Z = 0.000	1.000
	CD3+PD1+(%)	29.67 ± 4.45	31.12 ± 6.26	t = −0.660	0.515
	CD3^+^CD28+(%)	65.96 ± 13.14	64.96 ± 11.61	t = 0.208	0.837
	CD4+PD1+(%)	25.87 ± 5.66	29.08 ± 7.73	t = −1.173	0.252
	CD4^+^CD28+(%)	87.37 (80.16.90.23)	79.01 (70.36.89.04)	Z = −1.036	0.318
	CD8+PD1+(%)	34.30 ± 13.25	33.86 ± 10.69	t = 0.094	0.926
	CD8^+^CD28+(%)	43.02 ± 15.31	42.60 ± 12.94	t = 0.076	0.940
	CD3+PD1+	282.00 (206.00,310.00)	475.00 (186.50,715.50)	Z = −1.086	0.294
	CD3^+^CD28^+^	591.00 (396.00,822.00)	893.50 (387.50,1883.50)	Z = −1.184	0.251
	CD4+PD1+	139.00 (135.00,182.00)	226.50 (117.00,500.25)	Z = −1.481	0.148
	CD4^+^CD28^+^	462.00 (264.00,637.00)	693.50 (291.25,1597.25)	Z = −1.283	0.212
	CD8+PD1+	92.00 (64.00,117.00)	126.00 (51.25,214.00)	Z = −0.346	0.753
	CD8^+^CD28^+^	134.00 (99.00,151.00)	164.50 (63.50,396.50)	Z = −0.691	0.512
Post-treatment	Treg	4.27 ± 1.79	4.70 ± 2.00	t = 0.764	0.576
	CD3+PD1+(%)	0.65 (0.42.1.27)	0.63 (0.45.0.93)	Z = −0.025	0.981
	CD3^+^CD28+(%)	59.00 ± 9.70	70.00 ± 9.66	t = −2.896	0.008
	CD4+PD1+(%)	0.37 (0.28.0.57)	0.45 (0.26.0.74)	Z = −0.148	0.904
	CD4^+^CD28+(%)	73.41 (60.44.87.76)	85.90 (77.23.92.36)	Z = −2.073	0.039
	CD8+PD1+(%)	0.59 (0.56.1.27)	0.85 (0.57.1.26)	Z = −0.765	0.451
	CD8^+^CD28+(%)	43.32 ± 16.35	45.45 ± 14.14	t = −0.362	0.721
	CD3+PD1+	6.00 (3.00.10.00)	6.00 (3.24.9.75)	Z = −0.223	0.827
	CD3^+^CD28^+^	483.00 (361.00,649.00)	616.00 (339.25,916.75)	Z = −0.592	0.577
	CD4+PD1+	2.00 (2.00.3.00)	2.00 (2.00.4.75)	Z = −0.285	0.790
	CD4^+^CD28^+^	318.00 (270.00,482.00)	436.50 (292.25,734.75)	Z = = -1.160	0.251
	CD8+PD1+	2.00 (1.00.4.00)	2.00 (1.00.3.00)	Z = −0.504	0.645
	CD8^+^CD28^+^	134.00 (67.00,210.00)	99.00 (60.00,186.50)	Z = −0.839	0.422
% change	Treg	0.25 ± 0.48	0.32 ± 0.54	t = −0.337	0.739
	CD3+PD1+(%)	−0.98 (-0.98,-0.96)	−0.97 (-0.99,-0.96)	Z = −0.197	0.865
	CD3^+^CD28+(%)	−0.05 (-0.25.0.07)	0.07 (-0.04.0.25)	Z = −2.023	0.044
	CD4+PD1+(%)	−0.99 (-0.99,-0.98)	−0.98 (-0.99,-0.97)	Z = −0.148	0.904
	CD4^+^CD28+(%)	−0.08 (-0.29.0.03)	0.04 (-0.02.0.11)	Z = −2.171	0.030
	CD8+PD1+(%)	−0.98 (-0.98,-0.97)	−0.97 (-0.98,-0.95)	Z = −0.642	0.544
	CD8^+^CD28+(%)	0.03 (-0.12.0.07)	0.03 (-0.16.0.25)	Z = −0.296	0.790
	CD3+PD1+	−0.98 (-0.99,-0.94)	−0.98 (-0.99,-0.96)	Z = −1.036	0.318
	CD3^+^CD28^+^	−0.05 (-0.41.0.39)	−0.25 (-0.58,-0.08)	Z = −1.431	0.163
	CD4+PD1+	−0.99 (-0.99,-0.98)	−0.99 (-1.00,-0.97)	Z = −0.691	0.512
	CD4^+^CD28^+^	−0.19 (-0.42.0.36)	−0.19 (-0.60.0.18)	Z = −0.938	0.368
	CD8+PD1+	−0.98 (-0.99,-0.97)	−0.98 (-0.99.0.97)	Z = −0.667	0.512
	CD8^+^CD28^+^	0.12 (-0.40.0.60)	−0.44 (-0.67,-0.03)	Z = −1.900	0.056

### 4.4 Correlation analysis between composite response and peripheral blood immune cells in different treatment groups

**FIGURE 2:**
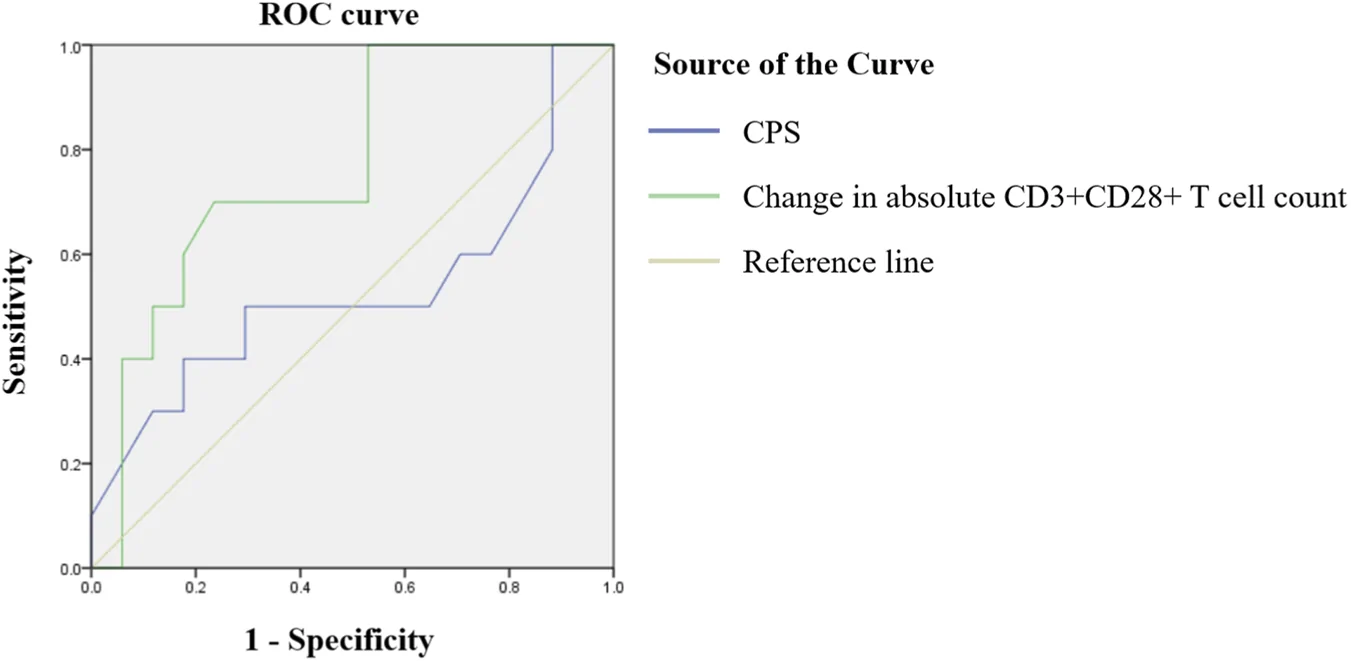
ROC curves for treatment efficacy plotted using the CPS score and the absolute change in CD3^+^CD28^+^ T cell count as test variables.

The pathological responses of the primary tumor and cervical lymph nodes were integrated for evaluation, with simultaneous achievement of pCR in both sites (dual pCR) defined as the optimal composite endpoint. In the chemoimmunotherapy group, the dual pCR rate was 37.0% (10/27). Subgroup analysis showed that the percentage of peripheral blood CD3^+^CD28^+^ cells remained at a low level after neoadjuvant treatment in the dual pCR group, with a significant difference compared with the non-dual pCR group (P = 0.006). Within-group comparison before and after treatment revealed a decreasing trend in the proportion of CD3^+^CD28^+^ cells in the dual pCR group; although the difference did not reach the conventional threshold of statistical significance (P = 0.052), taken together with the independent analyses of primary tumor and lymph node responses presented above, this trend was highly consistent with the overall pattern of peripheral blood activated T cells migrating into the tumor and lymph nodes during effective treatment (Table 5). ROC curves for predicting treatment response were constructed using the CPS score and the absolute change in CD3^+^CD28^+^ T cells as test variables. The area under the curve (AUC) for the absolute change in CD3^+^CD28^+^ T cells was 0.768 (95% CI: 0.584–0.951). At a Youden index of 0.471, the optimal cutoff value was −76.5, corresponding to a sensitivity of 100.0% and a specificity of 47.1% (Figure 2).

**TABLE 5:** Peripheral blood immune indices in the chemoimmunotherapy group by combined primary tumor and lymph node pathological response.

Category	Item	Chemoimmunotherapy		Statistical	*P*
		pCR (n = 10)	Non-pCR (n = 17)		
Pre-treatment	CPS	19.00 (4.75.80.00)	20.00 (5.50.40.00)	Z = −0.278	0.786
Pre-treatment	Treg	3.21 (2.95.4.16)	3.68 (2.55.4.66)	Z = −0.201	0.863
	CD3+PD1+(%)	28.86 ± 3.75	31.51 ± 6.27	t = −1.208	0.238
	CD3^+^CD28+(%)	65.68 ± 13.82	65.18 ± 11.28	t = 0.102	0.920
	CD4+PD1+(%)	78.67 ± 22.63	79.36 ± 13.00	t = −1.659	0.110
	CD4^+^CD28+(%)	87.25 (76.52.90.17)	79.86 (72.05.89.93)	Z = −0.703	0.505
	CD8+PD1+(%)	32.33 ± 12.16	35.04 ± 11.43	t = −0.581	0.566
	CD8^+^CD28+(%)	42.03 ± 15.76	43.21 ± 12.78	t = −0.212	0.834
	CD3+PD1+	280.00 (189.75,313.75)	474.00 (191.00,696.00)	Z = −1.205	0.243
	CD3^+^CD28^+^	599.00 (367.25,845.50)	780.00 (399.00,1846.00)	Z = −1.054	0.309
	CD4+PD1+	138.00 (117.00,180.00)	204.00 (118.00,480.50)	Z = −1.557	0.127
	CD4^+^CD28^+^	435.00 (253.75,682.50)	543.00 (292.50,1576.50)	Z = −1.305	0.204
	CD8+PD1+	91.00 (63.00,117.00)	150.00 (52.50,207.00)	Z = −0.603	0.570
	CD8^+^CD28^+^	132.50 (91.25,162.25)	151.00 (65.00,388.00)	Z = −0.854	0.414
Post-treatment	Treg	4.30 ± 1.88	4.66 ± 1.95	t = −0.461	0.649
	CD3+PD1+(%)	0.73 (0.41.1.29)	0.65 (0.47.0.92)	Z = −0.025	0.980
	CD3^+^CD28+(%)	58.22 ± 9.85	69.78 ± 9.39	t = −3.036	0.006
	CD4+PD1+(%)	0.36 (0.27.0.66)	0.45 (0.26.0.69)	Z = −0.402	0.711
	CD4^+^CD28+(%)	74.02 (60.37.88.63)	84.46 (70.94.92.10)	Z = −1.657	0.103
	CD8+PD1+(%)	0.59 (0.49.1.18)	0.86 (0.62.1.28)	Z = −1.130	0.264
	CD8^+^CD28+(%)	41.97 ± 16.58	46.12 ± 13.96	t = −0.697	0.492
	CD3+PD1+	7.00 (3.00.11.50)	6.00 (3.50.9.50)	Z = −0.404	0.711
	CD3^+^CD28^+^	586.80 ± 317.06	657.41 ± 344.88	t = −0.529	0.602
	CD4+PD1+	2.50 (1.75.3.00)	2.00 (20.00.4.50)	Z = −0.106	0.941
	CD4^+^CD28^+^	318.00 (292.75,517.25)	404.00 (269.00,725.50)	Z = −0.829	0.414
	CD8+PD1+	2.00 (0.75.4.75)	2.00 (1.00.3.00)	Z = −0.179	0.863
	CD8^+^CD28^+^	163.30 ± 129.11	124.06 ± 78.00	t = 0.992	0.330
% change	Treg	0.28 ± 0.50	0.31 ± 0.53	t = −0.144	0.887
	CD3+PD1+(%)	−0.98 (-0.99,-0.96)	−0.97 (-0.99,-0.97)	Z = −0.351	0.749
	CD3^+^CD28+(%)	−0.06 (-0.27.0.08)	0.06 (-0.04.0.23)	Z = −1.958	0.052
	CD4+PD1+(%)	−0.99 (-0.99,-0.97)	−0.98 (-0.99,-0.97)	Z = −0.151	0.902
	CD4^+^CD28+(%)	−0.07 (-0.22.0.03)	0.04 (-0.06.0.10)	Z = −1.657	0.103
	CD8+PD1+(%)	−0.98 (-0.99,-0.97)	−0.97 (-0.98,-0.96)	Z = −0.703	0.505
	CD8^+^CD28+(%)	0.03 (-0.12.0.08)	0.07 (-0.15.0.23)	Z = −0.452	0.675
	CD3+PD1+	−0.98 (-0.99,-0.93)	−0.98 (-0.99,-0.96)	Z = −1.155	0.264
	CD3^+^CD28^+^	0.12 (-0.45.0.40)	−0.24 (-0.57,-0.09)	Z = −1.406	0.170
	CD4+PD1+	−0.98 (-0.99,-0.98)	−0.99 (-0.99,-0.97)	Z = −0.854	0.414
	CD4^+^CD28^+^	−0.03 (-0.45.0.40)	−0.21 (-0.58.0.12)	Z = −1.205	0.243
	CD8+PD1+	−0.98 (-0.99,-0.96)	−0.98 (-0.99,-0.97)	Z = −0.729	0.473
	CD8^+^CD28^+^	0.18 (-0.42.0.61)	−0.41 (-0.64,-0.05)	Z = −1.833	0.066

### 4.5 Representative case analysis

**FIGURE 3:**
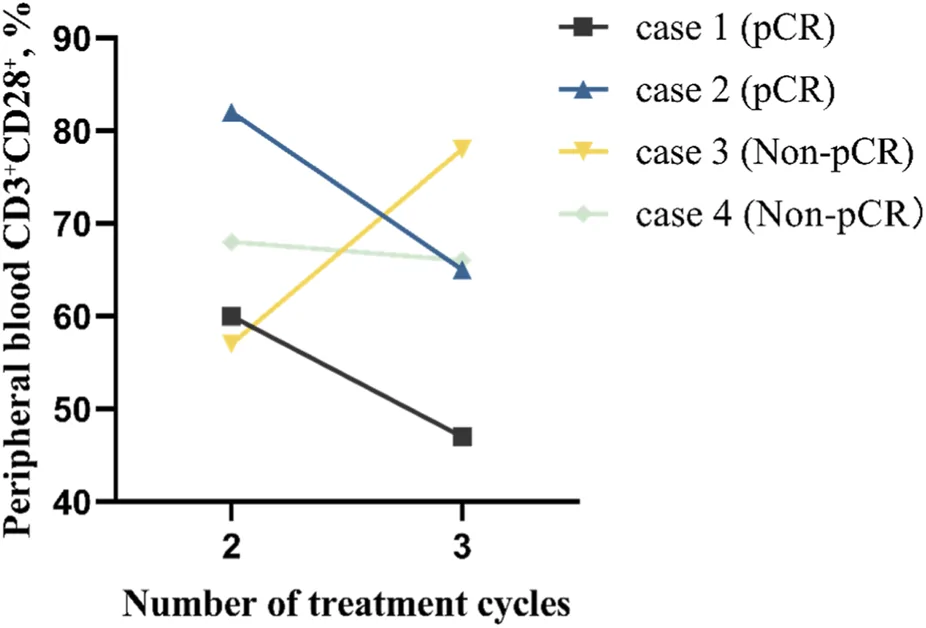
Changes in peripheral blood CD3^+^CD28^+^ percentage after chemoimmunotherapy in a representative case.

In the chemoimmunotherapy group, four patients were assessed as having a partial response (PR) by imaging after two cycles of neoadjuvant treatment, which was subsequently confirmed pathologically, and they received one additional cycle of the same regimen. Ultimately, two patients achieved dual pCR in both the primary tumor and lymph nodes after three cycles, while the other two remained in PR. Comparison of the dynamic changes in the percentage of peripheral blood CD3^+^CD28^+^ cells among these four patients revealed that those who ultimately benefited from treatment (dual pCR) exhibited a sustained and pronounced decreasing trend in this parameter (Figure 3, Cases 1–2), whereas those who did not achieve dual pCR showed relatively stable or even rebounding CD3^+^CD28^+^ cell proportions (Figure 2, Cases 3–4). These individual dynamic patterns were highly consistent with the group-level analysis results, further validating, from a temporal perspective, the potential of a decline in peripheral blood CD3^+^CD28^+^ cells as a surrogate marker of treatment response.

## 5 Discussion

In this study, through a retrospective analysis of 57 patients with locally advanced HNSCC, we systematically evaluated the correlation between peripheral blood T cell subsets and pathological response to neoadjuvant therapy. The results revealed that, following chemoimmunotherapy, decreases in both the absolute count and percentage of peripheral blood CD3^+^CD28^+^ cells were significantly associated with pathological complete response in the primary tumor, cervical lymph node metastases, and dual sites. In the chemotherapy group, this association was confined to primary tumor pCR and was comparatively weaker in magnitude. Moreover, neoadjuvant chemoimmunotherapy not only increased the pathological complete response rate but also significantly reduced the extent of subsequent surgical resection, thereby creating more favorable conditions for organ preservation. At the individual level, a sustained decline in the proportion of CD3^+^CD28^+^ T cells during treatment could distinguish patients who ultimately benefited from therapy in real time, highlighting its potential as a non-invasive dynamic monitoring biomarker. All subgroup analyses were conducted after confirming that baseline characteristics were balanced and comparable, in order to minimize potential confounding bias.

Data from several recently published phase II clinical trials have demonstrated that neoadjuvant chemoimmunotherapy can yield pCR rates as high as 30%–47.6% in resectable locally advanced HNSCC, significantly surpassing those achieved with immunotherapy alone or chemotherapy alone (Lv et al., 2022). In the present study, the primary tumor pCR rate in the chemoimmunotherapy group was 75.0% (24/32), with a dual pCR rate of 37.0%, which is broadly consistent with these previous reports. Furthermore, the reduction in surgical extent observed in the combination therapy group was concordant with the pathological response, lending further support to the important role of neoadjuvant chemoimmunotherapy in this patient population. However, even under the regimens yielding the highest pCR rates, nearly half of patients still fail to achieve a major pathological response; therefore, identifying reliable predictive biomarkers remains an urgent priority. In this study, CPS showed no statistically significant difference between the pCR and non-pCR groups, which is in line with the growing body of evidence indicating that tumor tissue-based PD-L1 expression is not an ideal predictor of treatment efficacy in the neoadjuvant immunotherapy setting (Cohen et al., 2019).

The central finding of this study is that the post-treatment decline in peripheral blood CD28^+^ T cell subsets is closely associated with pathological complete response. This phenomenon can be interpreted from the perspective of co-stimulatory signaling in T cell activation. CD28 is a critical co-stimulatory molecule for full T cell activation and plays an indispensable role in anti-PD-1 therapy: the re-expansion and restoration of effector function in exhausted T cells “rescued” by PD-1 blockade are strictly dependent on CD28 signaling (Kamphorst et al., 2017; Flemming, 2017; Krueger and Rudd, 2017). Therefore, the decrease in the proportion of CD3^+^CD28^+^ cells in peripheral blood may not reflect an absolute reduction in cell numbers due to apoptosis or exhaustion, but rather a “homing” process in which activated, antigen-specific T cells migrate from the circulation into tumor tissues (including the primary tumor and regional lymph nodes) and become retained and infiltrated there. Huang et al. (Huang et al., 2017) have previously described across multiple solid tumor types that the ratio of peripheral blood T cell reinvigoration to tumor burden can predict clinical benefit from anti-PD-1 therapy, the core of which lies in the rapid egress of activated T cells into the periphery. Wu et al. (Wu et al., 2020) demonstrated the expansion of tumor-infiltrating T cells following anti-PD-1 therapy. Yost et al. (Yost et al., 2019) further found that newly emerging intratumoral effector T-cell clones after PD-1 blockade originate predominantly from newly recruited T cells in the peripheral blood, rather than from local reinvigoration of pre-existing intratumoral T cells. Our results are consistent with this dynamic model and provide the first evidence, in the setting of neoadjuvant therapy for HNSCC, linking a post-treatment reduction in circulating activated T cells marked by CD28 with pathological complete response.

Notably, pathological response in lymph node metastases was associated not only with decreases in CD3^+^CD28^+^ and CD8^+^CD28^+^ T cells, but also with a concurrent decline in CD4^+^CD28^+^ T cells. As lymph nodes serve as hubs for the initiation and regulation of adaptive immune responses, the complete eradication of metastatic deposits within them may require stronger “help” signals provided by CD4^+^ helper T cells to sustain the infiltration and function of CD8^+^ effector T cells (Alspach et al., 2019). In the present study, no significant association was observed between lymph node response and peripheral blood immune cell changes in the chemotherapy group, suggesting that chemotherapy alone is insufficient to elicit adequate T cell migration and effector responses within the more profoundly immunosuppressive lymph node microenvironment. This is consistent with the concept of “systems immunity” proposed by Spitzer et al. (Spitzer et al., 2017), which emphasizes that effective anti-tumor immunity requires coordinated re-activation of the systemic immune system. The addition of immune checkpoint inhibitors, by synergizing with tumor antigens released by chemotherapy, amplifies a local immune perturbation into a systemic T cell mobilization, thereby enabling immune-mediated clearance in distant sites such as lymph nodes. Furthermore, the reduction in Treg cells observed in patients achieving primary tumor pCR in the chemotherapy group aligns with reports that taxanes and platinum agents can selectively suppress Tregs (Roselli et al., 2013); however, the intensity of such immunomodulation appears insufficient to overcome the immune-privileged status of lymph nodes.

Recent studies have underscored, from various dimensions, the necessity of integrating peripheral blood immune markers with features of the tumor microenvironment. Li et al. (Li et al., 2025) investigated peripheral immune and inflammatory markers for predicting neoadjuvant immunotherapy response in HNSCC and found that the combined analysis of circulating immune cells and inflammatory factors provides a more comprehensive reflection of the host antitumor immune status than any single parameter alone. Noda et al. (Noda et al., 2025) employed spatial transcriptomics and immunohistochemistry to reveal the spatially heterogeneous expression of immune checkpoint molecules such as B7-H4/VTCN1, suggesting that a single-time-point peripheral blood assay may not fully capture the complexity of tumor-immune interactions. Together, these advances indicate that peripheral blood T-cell dynamics should not be regarded as an isolated circulating phenomenon, but rather as a window into systemic immune reinvigoration that ultimately needs to be interpreted in the context of tumor tissue immune microenvironment characteristics. The association between the decline in CD3^+^CD28^+^ T cells and pathological response observed in this study, if integrated with paired tissue T-cell infiltration, spatial immune architecture, and functional status in future investigations, may help to more comprehensively elucidate its biological significance.

From a predictive performance perspective, the dynamic change in the percentage of peripheral blood CD3^+^CD28^+^ T cells demonstrated consistently robust predictive capability across different tumor sites. Notably, in the composite response analysis, the decreasing trend in the proportion of CD3^+^CD28^+^ cells in the dual pCR group reached borderline significance (P = 0.052), and the representative case analysis clearly illustrated the temporal correspondence between the decline of this marker at the individual level and ultimate treatment benefit. This finding suggests that early peripheral blood sampling during treatment (e.g., after 1-2 cycles) can identify patients who are likely to achieve dual pCR based on changes in the CD28^+^ T cell subset, thereby providing a real-time, non-invasive biological basis for individualized decision-making regarding the neoadjuvant treatment course, such as continuing the original regimen, timely transitioning to surgery, or switching to an alternative therapeutic strategy. Compared with CPS, which requires tumor tissue biopsy, peripheral blood-based biomarkers offer the inherent advantages of repeated accessibility, the ability to overcome tissue heterogeneity, and suitability for dynamic monitoring.

This study has several limitations. As a single-center retrospective study, the sample size is limited, particularly the small size of the dual pCR subgroup, which restricted statistical power and precluded multivariate analysis. In particular, the association analysis between CD3^+^CD28^+^ T cell dynamics and pathological response was primarily based on univariate comparisons. Although the influence of known clinical factors and inter-individual differences in baseline immune status was preliminarily controlled by confirming baseline balance, the potential interference of other unmeasured confounders cannot be completely excluded. Therefore, the independent predictive value of the CD3^+^CD28^+^ T cell change trend observed in this study needs to be validated in prospective, large-sample studies through multivariable regression analyses that incorporate additional potential variables (e.g., tumor mutational burden, inflammatory markers). Moreover, because paired immunohistochemistry or TCR analysis was not performed on post-treatment tumor tissue, the direct causal relationship between the decline in peripheral blood CD28^+^ T cells and increased intratumoral infiltration could not be directly demonstrated in the present study. Future investigations should incorporate concurrent sampling and paired analysis of peripheral blood and tumor tissue. Moreover, the retrospective design did not allow standardization of the blood sampling time window, and high-dimensional phenotypic or functional analyses of peripheral blood immune cells were not performed, potentially overlooking subsets of greater predictive value. Future prospective multicenter cohorts should establish fixed blood sampling time points before treatment, after each cycle, and before surgery, and use techniques such as mass cytometry, single-cell transcriptomics, or TCR tracking to further validate the predictive threshold of CD3^+^CD28^+^ T cell dynamics and explore their intrinsic relationship with immune infiltration within the tumor microenvironment.

In conclusion, this study demonstrates that neoadjuvant chemoimmunotherapy can induce a significant decrease in peripheral blood CD28^+^ T cell subsets in patients with HNSCC, and this dynamic change is closely associated with pathological complete response in the primary tumor, lymph nodes, and dual sites, with superior predictive performance compared with traditional tissue-based PD-L1 scoring. Monitoring the migratory response of peripheral blood activated T cells holds promise as a novel non-invasive strategy to guide individualized neoadjuvant therapy in locally advanced HNSCC.

## Data Availability

The original contributions presented in the study are included in the article/Supplementary Material, further inquiries can be directed to the corresponding author.
