# Supplementary material for: Analysis of peripheral blood T-cell levels for predicting immunotherapy response sensitivity in squamous cell carcinoma of the head and neck
Source: Front Mol Biosci. 2026 Aug 6;13:1896236. doi: 10.3389/fmolb.2026.1896236 (PMC13489828; doi:10.3389/fmolb.2026.1896236)
Supplement: Supplementary file 1 [file Table1.docx]

Supplementary Table 1. Peripheral blood immune indices in the chemotherapy group by lymph node pathological response.

| Category | Item | Chemotherapy | | Statistical | *P* |
| --- | --- | --- | --- | --- | --- |
|  |  | pCR（n=7） | Non-pCR(n=13) |  |  |
| Pre-treatment | CPS | 8.00(3.00,35.00) | 30.00(6.50,60.00) | Z=-1.315 | 0.211 |
| Pre-treatment | Treg | 3.67(2.91,5.24) | 3.62(2.88,4.28) | Z=-0.040 | 1.000 |
|  | CD3+PD1+(%) | 32.06±7.21 | 29.21±7.76 | t=0.803 | 0.432 |
|  | CD3+CD28+(%) | 58.09±13.18 | 65.35±15.75 | t=-1.036 | 0.314 |
|  | CD4+PD1+(%) | 30.08±6.64 | 28.82±7.99 | t=0.355 | 0.727 |
|  | CD4+CD28+(%) | 82.32(67.29,83.86) | 84.63(72.27,90.09) | Z=-1.783 | 0.081 |
|  | CD8+PD1+(%) | 34.62±8.48 | 32.28±12.94 | t=0.429 | 0.673 |
|  | CD8+CD28+(%) | 30.15±15.26 | 43.13±17.60 | t=-1.643 | 0.118 |
|  | CD3+PD1+ | 451.14±297.90 | 247.85±85.45 | t=1.767 | 0.124 |
|  | CD3+CD28+ | 817.71±499.31 | 556.23±206.07 | t=1.326 | 0.226 |
|  | CD4+PD1+ | 296.57±215.21 | 145.85±46.06 | t=1.831 | 0.115 |
|  | CD4+CD28+ | 706.57±467.06 | 423.92±164.03 | t=1.550 | 0.166 |
|  | CD8+PD1+ | 129.43±68.35 | 92.54±56.06 | t=1.302 | 0.209 |
|  | CD8+CD28+ | 136.00(27.00,154.00) | 94.00(70.00,148.00) | Z=-0.555 | 0.588 |
| Post-treatment | Treg | 3.36(2.94,5.67) | 3.11(2.17,3.47) | Z=-1.190 | 0.241 |
|  | CD3+PD1+(%) | 34.76(25.67,46.77) | 32.24(24.85,35.57) | Z=-1.070 | 0.311 |
|  | CD3+CD28+(%) | 66.12±20.44 | 64.65±15.78 | t=0.181 | 0.858 |
|  | CD4+PD1+(%) | 33.51±9.81 | 27.12±9.97 | t=1.373 | 0.187 |
|  | CD4+CD28+(%) | 84.23(71.69,90.26) | 80.41(77.27,86.78) | Z=-0.515 | 0.643 |
|  | CD8+PD1+(%) | 34.64±10.29 | 32.01±12.88 | t=0.465 | 0.647 |
|  | CD8+CD28+(%) | 43.52±22.80 | 39.76±22.17 | t=0.358 | 0.725 |
|  | CD3+PD1+ | 225.00(165.00,1719.00) | 225.00(119.00,289.50) | Z=-1.110 | 0.275 |
|  | CD3+CD28+ | 505.00(211.00,2216.00) | 446.00(301.50,623.00) | Z=-0.753 | 0.485 |
|  | CD4+PD1+ | 174.00(106.00,1221.00) | 121.00(77.00,167.50) | Z=-1.466 | 0.157 |
|  | CD4+CD28+ | 412.00(225.00,1839.00) | 306.00(250.50,482.50) | Z=-1.070 | 0.311 |
|  | CD8+PD1+ | 82.00(51.00,352.00) | 55.00(27.50,117.00) | Z=-1.228 | 0.241 |
|  | CD8+CD28+ | 166.57±143.63 | 79.23±55.93 | t=1.547 | 0.166 |
| % Change | Treg | -0.10(-0.35,0.17) | -0.25(-0.57,-0.02) | Z=-1.228 | 0.241 |
|  | CD3+PD1+(%) | 0.10±0.24 | 0.02±0.27 | t=0.424 | 0.676 |
|  | CD3+CD28+(%) | 0.01(-0.03,0.18) | 0.03(-0.19,0.14) | Z=-0.594 | 0.588 |
|  | CD4+PD1+(%) | 0.14(-0.09,0.23) | 0.03(-0.19,0.12) | Z=-0.991 | 0.351 |
|  | CD4+CD28+(%) | 0.02(-0.13,0.28) | -0.04(-0.11,0.24) | Z=-0.357 | 0.757 |
|  | CD8+PD1+(%) | 0.01±0.22 | 0.01±0.37 | t=0.050 | 0.961 |
|  | CD8+CD28+(%) | 0.28(-0.01,0.47) | 0.01(-0.39,0.23) | Z=-1.704 | 0.097 |
|  | CD3+PD1+ | 0.25±0.73 | -0.11±0.43 | t=1.405 | 0.177 |
|  | CD3+CD28+ | 0.37±0.94 | -0.14±0.35 | t=1.385 | 0.209 |
|  | CD4+PD1+ | 0.37±0.80 | -0.12±0.37 | t=1.547 | 0.163 |
|  | CD4+CD28+ | 0.41±1.06 | -0.05±0.47 | t=1.082 | 0.314 |
|  | CD8+PD1+ | 0.09±0.79 | -0.22±0.42 | t=1.263 | 0.223 |
|  | CD8+CD28+ | 0.76±1.17 | -0.32±0.32 | t=2.382 | 0.051 |
