# Supplementary material for: Analysis of peripheral blood T-cell levels for predicting immunotherapy response sensitivity in squamous cell carcinoma of the head and neck
Source: Front Mol Biosci. 2026 Aug 6;13:1896236. doi: 10.3389/fmolb.2026.1896236 (PMC13489828; doi:10.3389/fmolb.2026.1896236)
Supplement: Supplementary file 2 [file Table2.docx]

**Supplementary Material: Flow Cytometry Protocol for Peripheral Blood Immune Cell Subset Detection**

**Antibodies and Reagents**

The following fluorochrome-conjugated monoclonal antibodies were used: CD3-PerCP, CD45-PE-Cy7, PD-1/CD279 (type A-FITC; type B-PE; type C-APC; type D-PerCP; type E-APC-Cy7; type F-Cy7), CD28-FITC, CD4-APC, and CD8-APC-Cy7, all purchased from BD Biosciences. Isotype controls were used in parallel.

**Staining and Acquisition**

Fresh EDTA-K2-anticoagulated whole blood (100 μL) was incubated with the antibody cocktail at room temperature in the dark for 20 min. Red blood cells were lysed with 1× FACS Lysing Solution (BD), washed with PBS, and resuspended in 300 μL sheath fluid. Samples were acquired on a BD FACSCanto II flow cytometer. A total of 5,000 events were collected within the lymphocyte gate for each sample.

**Gating Strategy**

Lymphocytes were gated on an FSC-A/SSC-A dot plot. Within the lymphocyte gate, CD3+ T cells were identified by gating CD3 versus SSC-A. CD4+ and CD8+ T cells were further discriminated by back-gating on CD4 and CD8. The expression percentages of CD28 and PD-1 were analyzed in each subset. Treg cells were defined as CD4+CD25highCD127low cells: within the CD4+ T-cell population, cells with high CD25 expression and low CD127 expression were selected, and their percentage among CD4+ T cells and absolute counts were recorded.

**Absolute Count Calculation**

The absolute count of each subset was calculated using a dual-platform method: Absolute subset count (cells/μL) = (subset percentage obtained by flow cytometry) × (absolute lymphocyte count from concurrent clinical complete blood count, cells/μL).

**Quality Control**

Unstained controls, single-stain compensation controls, and fluorescence-minus-one (FMO) controls were included in each experiment to ensure accurate gating. The laboratory participates in the external quality assessment program for flow cytometry organized by the National Center for Clinical Laboratories. All samples were processed within 24 hours of blood collection. The normal reference ranges were established from peripheral blood samples of 30 age-matched healthy volunteers and are presented in Supplementary Table 2.

Supplementary Table 2. Peripheral blood immune cell parameters and reference ranges.

| Test item | Reference range | Unit |
| --- | --- | --- |
| Treg (CD4+CD25+127LOW) | - | % |
| CD3+PD1+% | 0-40.82 | % |
| CD3+CD28+% | 32.13-79.33 | % |
| CD4+PD1+% | 0-42.10 | % |
| CD4+CD28+% | 48.93-100 | % |
| CD8+PD1+% | 0-41 | % |
| CD8+CD28+% | 14.55-73.11 | % |
| CD3+PD1+Absolute count | 0-573 | cells/μL |
| CD3+CD28+ Absolute count | 273-1090 | cells/μL |
| CD4+PD1+ Absolute count | 0-269 | cells/μL |
| CD4+CD28+ Absolute count | 161-746 | cells/μL |
| CD8+PD1+ Absolute count | 0-194 | cells/μL |
| CD8+CD28+ Absolute count | 47-344 | cells/μL |
